# Advancing positive social determinants of health through collective impact and the 100% New Mexico Model

**DOI:** 10.1186/s13690-023-01120-4

**Published:** 2023-06-16

**Authors:** Julie S. McCrae, Angeline K. Spain

**Affiliations:** Chapin Hall at the University of Chicago, Chicago, IL USA

**Keywords:** Collective impact, Social determinants of health, Adverse childhood experiences, Collaboration

## Abstract

**Background:**

Communities across the U.S. and globally confront the challenge of transforming negative social determinants of health (SDOH) into positive ones. To address this complex social problem, the collective impact (CI) approach has promise but has been critiqued for insufficiently challenging structural inequities. Research applying CI to SDOH is limited. This mixed-methods study examined early adoption of CI in the 100% New Mexico initiative that aims to address SDOH population-wide in a state with strong cultural identity and assets but also persistent socio-economic inequality.

**Methods:**

A web-based survey, interviews and focus groups were conducted with initiative participants in June and July 2021. Survey participants rated agreement on a 4-point scale with six items assessing CI foundation adapted from the Collective Impact Community Assessment Scale. Interviews and focus groups centered on motivation to engage, progress achieved in model components, CI core conditions, and contextual factors influencing experiences. Surveys were analyzed using descriptive means and proportions. Qualitative data were analyzed using thematic analysis and an inductive approach followed by stratified analyses and co-interpretation of emergent findings with model developers.

**Results:**

Fifty-eight participants completed the survey, and 21 individuals participated in interviews (n = 12) and two focus groups (n = 9). Survey mean scores were highest related to initiative buy-in and commitment, and lower related to shared ownership, having multiple perspectives and voices involved, and adequate resources. Qualitative results showed that the framework’s cross-sector emphasis helped motivate participation. Participants embraced the focus on leveraging existing community assets that is characteristic of CI and the current framework. Counties implemented effective engagement and visibility strategies including mural projects and book clubs. Participants expressed communication challenges across county sector teams which influenced feelings of accountability and ownership. Participants did not report challenges lacking relevant, available, and timely data or tension between funder-driven and community-driven desired outcomes, in contrast with previous CI research.

**Conclusion:**

Multiple foundational conditions of CI were supported in 100% New Mexico, including evidence for support of the common agenda addressing SDOH, shared measurement framework, and mutually reinforcing activities. Study results suggest that efforts to launch CI to address SDOH, which is by nature multi-sector, should include robust strategies to address communication needs of local teams. The use of community-administered surveys to identify gaps in SDOH resource access contributed to ownership and a sense of collective efficacy that may portend sustainability; however, relying on volunteers in the absence of other resources extensively also threatens sustainability.

**Supplementary Information:**

The online version contains supplementary material available at 10.1186/s13690-023-01120-4.

## Background

Research increasingly demonstrates the complexity in the social determinants of health over the life course. Health development is a complex interplay of biology and epigenetics, and one’s environment at the family, community, and system levels that interact throughout the life course to influence health [1]. There are differences in the likelihood of health (e.g. birth outcomes, communicable and noncommunicable diseases, and mental health) that are primarily due to social factors [2–4]. Considered social determinants of health (SDOH), these are the conditions where people are born, live, learn, work, play, worship, and age that affect a wide range of health, functioning, and quality of life outcomes and risks [5], and thus are very important to address effectively. Transforming adverse social determinants of health into positive ones lies in diverse service sectors across community environments, requiring a systemic, collective, and long-term approach.

There is no common definition of SDOH, nor a common research framework or evidence for how to address SDOH as a multi-faceted problem. The strategy explored in this article elevates a solution that is centered in addressing structural inequality and operationalizes positive SDOH as access to ten vital services necessary for surviving and thriving, a framework based on socio-ecological theory developed by the Anna, Age Eight Institute in 2018 [6]. Survival services are food, housing, medical/dental care, behavioral health care, and transportation. Thriving services are parent supports, early childhood learning programs, fully resourced community schools, youth mentor programs, and job training [7]. The *100% New Mexico* initiative, based on the 100% Community Model [7] assesses access to each of the ten service sectors within a community, disaggregates data to understand disparities, and engages a wide range of community partners to address a common agenda of transforming negative SDOH and experiences of adversity into positive SDOH and health. The approach uses collective impact [8]. This study examines the application of collective impact (CI) core conditions among eight counties that were early adopters of *100% New Mexico*. The study uses mixed methods, developmental evaluation including a participant survey, interviews and focus groups.

### Collective impact

The collective impact approach was specifically designed for complex social conditions. Originally developed in response to a lack of system-wide progress in U.S. education, collective impact refers to, “the commitment of a group of important actors from different sectors to a common agenda for solving a specific social problem” [8]. It is described as distinct from other multi-sector collaborations by nature of having centralized infrastructure, dedicated staff, and a structured process that is mutually reinforcing in aligning, re-routing or re-investing resources or scaling what already works [8]. Its seminal illustration, Strive (now StriveTogether) by 2019 had been implemented in 68 U.S. communities, with one-quarter to one-half showing improvements in reducing primary education disparity gaps [9].

There have been two primary critiques of collective impact. First, the model has insufficiently challenged structural inequity and is more top-down in its approach than harnessing the depth, value, and interests of community members [10, 11]. Second, its focus on collaboration locally has been thought to contribute to shifting responsibility for deep, systematic change away from government systems while at the same time under-resourcing fragmented sectors to implement the approach [12]. In 2022, the model is continuing to evolve, with its most robust attention to addressing equity, justice, and inclusion [13, 11]. To this end, communities implementing CI in the U.S. and internationally have led the effort to understand and resolve limitations of CI related to equity. CI efforts integrating equity center on shifting power, building equity leadership and accountability, using data to target solutions, acting with the community, and focusing clearly on systems change [11]. These five strategies for centering equity now complement CI’s original five essential conditions of a common agenda, shared measurement, mutually reinforcing activities, continuous communication, and a backbone team [8].

As is characteristic of many new approaches, CI, with eleven years in its history at this writing, lacks robust research evidence. Ennis and Tofa (2020) [10] conducted a systematic review of peer-review research of CI from 2011 to 2017. In 19 studies reviewed, just two reported on impacts. The remaining provide an implementation narrative drawn from participant perspectives and, to a more limited degree, document analysis. The results indicate strengths of CI in its adaptability to different contexts and issues and being able to layer the framework onto existing strategies. Across studies, challenges include achieving data sharing, linking, and use, and the potential harm within this to have smaller or less sophisticated community organizations further marginalized [10]. Limited resources overall, which often favors larger organizations, represents another challenge [14]. It is clear that specific, resourced attention to inclusion and addressing structural inequities will be important to address in CI going forward.

Outcome studies include a national study of six U.S. Healthy Start CI Peer-Learning Networks [15] and an Australian study that compared costs and output between CI and an alternative model to oral health care in rural Aboriginal communities [16]. Both studies showed positive results. Healthy Start CI participants reported increased skills and knowledge in CI and most reported that the approach had an impact on five of eight desired outcomes including improving processes and navigation that supports families’ access to comprehensive care, mobilizing the community, and increasing capacity to address social determinants of health [15]. The approach was rated less favorably for three areas of impact—integrating consumers into the planning of services; data systems; and family stability. Gwynne and Cairnduff’s [16] 2-year study found that costs were significantly lower (by 25%) with a CI service delivery model compared with services as usual that involved bringing in outside community resources. The study found that patients in the CI model received 47% more treatment than patients in the comparison condition. Authors attributed the differences to CI’s emphasis on drawing on the resources existing within the community and directing strategies to facilitate patients’ access to services, for example, publishing a service directory that included service hours, and offering services in multiple, convenient locations [16].

### 100% New Mexico collective impact framework

**Fig. 1:**
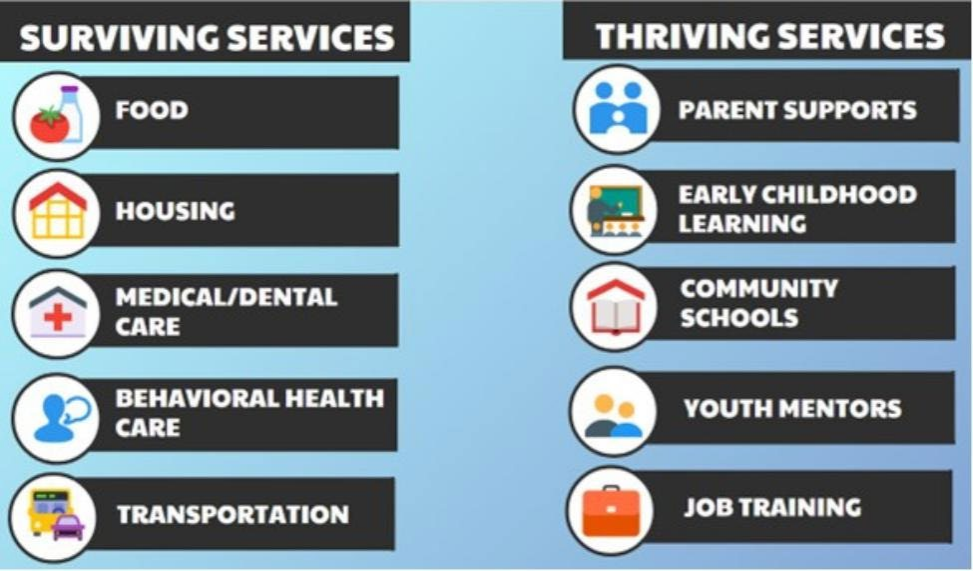
Sector Action Teams

In 2019, the Anna, Age Eight Institute (AAEI) was formed as a higher education-sponsored program with the mission of ensuring that every family in New Mexico has equal opportunity to thrive (https://annaageeight.nmsu.edu/), with access to ten vital services to address adverse childhood experiences (ACEs), family trauma, and adverse social determinants of health. Based on social-ecological theory and a public health approach, the AAEI developed and launched the *100% New Mexico* initiative, which has at its base a CI data-driven strategy meant to accelerate long-term, community-driven systems change. Within a county-based initiative, local representatives from these ten surviving and thriving services (Fig. 1) are organized as local Sector Action Teams that form the structure to implement CI, guided by the four-step phase of continuous quality improvement (CQI): assessment, planning, action, and evaluation focused on removing barriers to services.

**Fig. 2:**
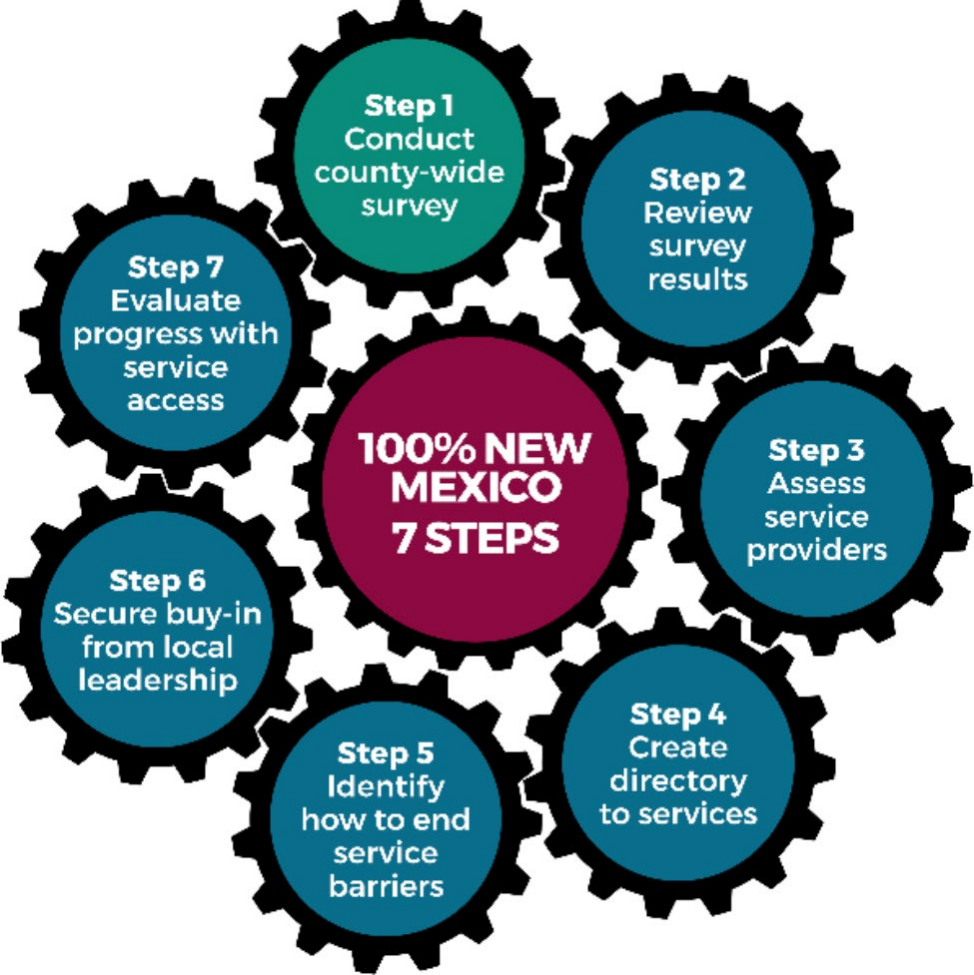
7-step implementation process of the 100% New Mexico framework *Courtney & Cappello, 2018*

Counties voluntarily participate in *100% New Mexico*, first contacting AAEI, inviting AAEI developers to facilitate presentations to local stakeholders, participating in training on CI and CQI, and beginning local planning to identify evidence-informed solutions guided by seven model steps (Fig. 2). The first step is to conduct a county-wide survey that asks community members about their need for each vital service, whether they have had difficulty accessing each service, and if so, the reason/s for their difficulty accessing the service. The initiative was explicitly designed to be tailored by each county, to respond to different values related to community mobilizing, beliefs about the role of local government in prioritizing and funding vital services, and their own community histories of trauma and service disparities.

## Methods

This study examines the development of core CI conditions as part of counties’ *100% New Mexico* initiative implementation. Developmental evaluation was used in this study phase as it is intended to support program decision makers’ strategic learning about program development and guide adaptation to local community contexts [17]. Developmental evaluation is well suited to the early stages of CI, where implementers are still defining how they will measure progress and evaluate effectiveness and impact [18]. Research shows that affecting systems change related to health involves financial resources, interorganizational relationships, involvement of the public health agency, and political relationships [19]. Yet there is limited understanding of the processes to systematically foster the core conditions of CI, particularly to bolster positive SDOH.

To address this gap, this investigation employs a concurrent mixed methods design to describe CI core conditions among a set of early adopter counties. New Mexico is a largely rural state and the eight early adopter counties included two rural, four mixed urban and rural, and two that included metropolitan or small metro areas. The interdisciplinary research team included three individuals with a mix of graduate training and research experience in psychology, education, and social welfare and one individual with experience delivering youth-focused prevention services. We describe results of a survey administered to *100% New Mexico* participants, then examine counties’ experiences developing local initiatives. Research questions are: (1) What motivated participation in the local CI initiative?; (2) What was the role of cross-sector communication in the first implementation year?; (3) What progress was made in mindset shifts and action to address identified needs (as compared with problem identification)?; and (4) What do participants report about inclusivity and partnership in the long-term CI work?

### Survey sample

The survey was distributed by county leads to all individuals participating in *100% New Mexico* in June 2021. In total, 69 individuals accessed the survey, of whom 66 consented. Of 66, 10 did not complete any items, resulting in a final sample of 58 responses. At survey distribution, most of the 7 counties for survey distribution were 6 to 12 months into implementation.

Participants represented a range of sectors: non-profit (23%); state government (21%); city government and higher education (9% each), private sector (4%), and county government (2%). Remaining participants did not indicate a sector (17%) or were classified as other (15%). Participants’ Sector Action Teams were community schools (22%), behavioral health (19%), early childhood education (19%), housing (9%), medical care (9%), mentoring (9%), parent supports (6%), food, transportation (3% each), and job training (1%).

### Interview and focus group sample

Over the same time period, we recruited the local CI champions in seven counties to participate in interviews. In an eighth county we conducted a qualitative case study that did not include the CI survey because they were undertaking the county-wide survey at the time. In this county, we invited the local CI champion and five individuals serving on the Core Team (who also led Sector Action Teams) to participate in interviews and members of two additional Sector Action Teams to participate in focus groups.

### Data collection

Survey participants rated six items on a 4-point scale: (1) do not agree, (2) agree a little, (3) agree somewhat, or (4) agree a lot. Items were based on the Collective Impact Community Assessment Scale as indicators of CI foundation [20]. This is a scoring matrix that was designed to assess CI initiatives on 14 core CI dimensions from establishing a common agenda to achieving systems change in advocacy and public policy. In its typical use, CI initiatives are rated on a 9-point scale from no impact to high impact drawing from multiple data sources. For this study, we designed a survey to include indicators of core CI conditions suitable for an emerging initiative. CI items assessed shared commitment to mission, shared commitment to goals, shared ownership for follow-through, resource adequacy, inclusivity, and that the right core partners are involved to make an impact. The scale demonstrated high internal consistency (α = 0.87).

We conducted 45-minute semi-structured interviews with CI champions in seven counties (*n* = 8 total, two co-leads were interviewed in one county). In the case study county, we conducted 45-minute interviews with the CI champion and Sector Action Team leads (*n* = 4) and 1-hour focus groups with two Sector Action Teams (*n* = 2, 9 participants total). Importantly, our qualitative data collection included the time spanning the 2020–2021 COVID-19 pandemic, allowing us to explore the framework’s applicability during a public health emergency. Interview and focus group instruments addressed the following: motivation to engage with *100% New Mexico*, progress achieved by their Sector Action Team and across sectors, progress fostering CI core conditions, and contextual factors influencing their experiences.

### Data analysis

Descriptive analyses using means and proportions were used for survey results. For qualitative data, we employed a codebook approach to thematic analysis [21]. A team of two researchers generated initial codes drawn from the three main topic areas for interviews and focus group topics and used Atlas.ti 8 qualitative analysis software to code and search for themes related to CI core conditions and contextual factors. We refined the themes using the constant comparative method, exploring alternative interpretations using matrices (by sector and by county) and comparison with survey findings. A single researcher then recoded the data to systematically capture contextual factors related to mindset shifts and the volunteer nature of the local CI initiatives [22]. To check the trustworthiness of the analysis process, we met separately with the model developers and local CI champions, incorporating in particular feedback about capacity and resources for local CI initiative implementation.

## Results

Study participants reported embracing *100% New Mexico’s* community-led approach which allowed counties to leverage existing strengths while also having 100%’s guiding framework and the focus on data drawn from community members to understand local service gaps and the reasons behind them. Participants reported that the flexibility supported by the model was critical to engaging key community leaders and sectors effectively and developing their initiatives in ways that incorporated local context and assets. The *100% New Mexico* model’s emphasis on vital services, which represent critical positive SDOH, resonated strongly during the COVID-19 pandemic and economic crisis. Initiative members indicated that this framework supported their efforts to conceptualize and coordinate community COVID-19 emergency responses.

### Collective impact survey results

**Fig. 3:**
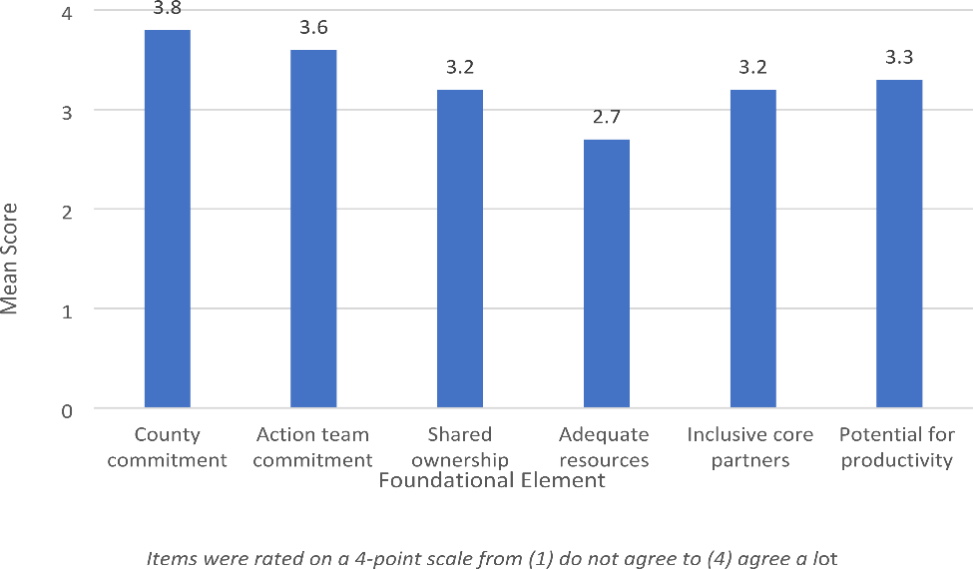
Respondent’s agreement with six foundational CI elements (n = 58)

Analyses of survey, interview, and focus group data indicated high levels of commitment to the local CI initiative. Mean scores on the six CI survey items are presented in Fig. 3.

Participants reported the highest agreement that a core group of partners in their county is committed to making a measurable impact (M = 3.8, SD = 0.54), and that their action team is committed to building greater alignment and connection to ensure service access (M = 3.6, SD = 0.73). Agreement was lower that their action team had the shared ownership needed to follow through on projects (M = 3.2, SD = 0.78), that the right core partners were involved to ensure multiple perspectives and voices (M = 3.2, SD = 0.74), and that the right core partners were involved to make progress to ensure service access (M = 3.3, SD = 0.80). Participants reported the lowest level of agreement that their action team had the resources needed to follow through on projects (M = 2.7, SD = 0.78).

The following present results of interviews and focus groups structured according to our research questions, specifically: (1) generating motivation to participate; (2) role of cross-sector communication, ownership, and accountability; (3) progress in mindset shifts and action to address identified needs; and (4) inclusivity and partnership.

### Generating motivation to participate

The common agenda component of CI is defined by having a shared vision for change, a common understanding of the problem, and a joint approach for problem-solving. Interview and focus group participants from the eight counties consistently emphasized the model’s focus on ten vital services and on action together as helping motivate their commitment to the vision at the county and Sector Action Team levels. One participant explained, “People who need food, they often need other things: like housing, like behavioral health, like childcare. It’s compounding [problems]…this is going be effective because it’s compounding help” (County 2). This reflects CI’s mutually reinforcing essential component. The model’s intentionality about reaching across sectors, coupled with specific action steps to address family burden of navigating a decentralized system of services, also motivated participants. Participants embraced the vision of building a family-centered service approach that is more responsive to the community.

Local CI initiative members used two specific experiential, community-building and knowledge-building strategies—book clubs and mural projects—that motivated their community members to convene around a shared vision. These strategies spread quickly to other counties. Some participants described being inspired by reading *Anna, Age Eight: The data-driven prevention of childhood trauma and maltreatment* and/or *100% Community: Ensuring 10 vital services for surviving and thriving* then starting a book club as a catalyst to bring others together to discuss their county-wide survey data. They noted that the two books differed in their purpose; *Anna, Age Eight* was a quick, compelling read that helped motivate the cross-sector focus that is important to the issue, while *100% Community* offered specific steps for moving into implementation. Public awareness events, for example, 100% Mural projects, were described as emerging for two reasons—as a way to recruit for Sector Teams, and to create visibility and engagement through expressive arts, which participants reported would resonate with community residents passionate about the issue, but not as interested in a book club. One explained, “That’s what invigorated me…that we were going to do something that was risky and fun. I think the energy just compounded after that” (County 3).

Participants reported that differences in the extent of Sector Action Teams’ existing networks posed a challenge to mobilizing more widespread interest. Particularly, early childhood learning and behavioral health teams often had strong existing coalitions, while others were establishing new partnerships. However, participants also noted that some Sector Action Teams with established networks also reported less robust progress and participation, suggesting that multiple factors played a role in achieving initial momentum.

### Role of cross-sector communication, ownership, and accountability

Implementing local *100% New Mexico* initiatives bolstered counties’ cross-sector communication and use of continuous improvement strategies overall, but there was varied progress. In this section, we discuss how these growth areas contributed to greater coordination across a range of activities, including both those led by local CI initiative members and by existing local coalitions.

*Cross-sector communication.* Continuous communication in CI is characterized as having consistent and open communication across participants to build trust and create common motivation. Participants viewed the strong communication within their own Sector Teams as an achievement, but also indicated the need for greater knowledge about other teams’ activities. In most counties, communication improved over time. They highlighted that despite communication challenges, the initiative was helping them better understand what other community agencies were doing and find areas of commonality—which were important early benefits. An interviewee noted, “I don’t think [before] we had a lot of opportunity to come to the table and say, ‘How can I help you in your challenge, and how can you help me?’” (County 1). Participants also stressed the value of cross-sector planning, supported by the *100% New Mexico* framework, to amplify outreach about events like hot meals and personal protective equipment giveaways during the COVID-19 pandemic.

*Ownership and accountability.* Participants stressed that the community-based coalition approach has helped foster local ownership. Agenda-setting guided by the community’s strengths and needs, rather than funder interests, was an asset. One interviewee recalled, “We had just done our health council priorities and we thought ‘Okay, here’s actually something– *an initiative–*that we could take on, that hits our priority areas [and] focuses on the one that the community told us that they want … most” (County 6). The community-led approach meant that counties had flexibility to prioritize concerns that were most pressing locally, as well as open to community leadership and interpretation, which was particularly important in more rural counties that tended to be very critical of outsiders. Without this community-led approach, participants noted it was unlikely that the local CI initiative would have been much different from other efforts led and defined by external groups that had ultimately failed to achieve envisioned changes.

The community-based coalition approach however, also meant that the local CI initiatives did not share a common backbone structure. They were housed at institutions of higher education, local public health councils, and mayor’s offices, among others and frequently with two or three co-chairs of the Core Team. Sector Team members reported being unclear about supports they could expect from the backbone structure as they moved to action. For example, they expressed that it could be difficult to understand who was accountable for doing what part of a project. A focus group participant explained, “One of the challenges at the beginning [was] ‘Where do we fit it?’…trying to identify what is a [Sector Action Team] role” (County 4). Limited communication across sectors and initiative participants also had contributed to frustration and losses of momentum at points for some communities: “People that are participating are not feeling like there is anything happening and that’s frustrating, right?” (County 7).”

### Progress in mindset shifts and action to address identified needs

In this section, we discuss mindset shifts and action taken to address identified needs. We describe the role of the local backbone infrastructure as a facilitator and barrier to ongoing progress across counties.

As shown in Table 1, the eight early adopter counties made demonstrable progress developing their local CI initiative during the first year, including administering the county-wide survey.

**Table 1:** Actions undertaken by early adopter counties in Year 1

Action	Number of counties
Held public engagement and initiative recruitment activities	8
Conducted county-wide survey	8
Engaged local CI initiative members to review assessment results	7
Developed and published a cross-sector resource directory	4
Developed local government buy-in for the initiative’s mission	1

Several of the urban and mixed urban/rural counties had strong, existing coalitions or prior experience attempting to address community service access; this generally contributed to readiness for change and progress. For example, in some counties, many members of the local CI initiative had been and continued to be involved in their local public health planning councils and they brought these connections and knowledge to the current CI initiative. However, individuals that were part of existing structures could also be reluctant to engage with the local CI initiative because they perceived it as failing to sufficiently value other efforts. Circumstances of limited capacity and scarce resources contributed to skepticism that adopting the *100% New Mexico* framework would result in change.

Champions and others that became involved early on reported that strategies like individual outreach and incorporating leadership of existing structures into local CI initiatives helped navigate these contextual factors. Many emphasized that the local CI champion’s passion and commitment were major strengths playing a role in their and others’ engagement: their “vision for the group and having the professional relationships with individuals in all those sectors…is really what brings the opportunity to the table” (County 1). In one county, initial engagement challenges decreased when an individual who was well-known and from the community took on the CI champion role. Participants across the counties observed that many of their local CI champions and Core Team members were recognized advocates and mentors. This reinforces the importance of relationships and social capital in building momentum in local contexts where “politics come into play all the time—there’s always somebody angling for something” (County 4). Interviewees across counties discussed the importance of recognizing their community’s history (rather than dismissing it) in their coalition-building.

With respect to mindset shifts and action to identify needs, participants emphasized that the 100% Community book and materials, with clear action steps, timelines, and priorities were critical. One leader drew a contrast between how their community had moved forward after a 100% Community book club and how quickly the momentum stalled after an earlier Anna, Age Eight book club. In other counties, local champions’ community knowledge and their skill communicating how the *100% New Mexico* framework offered solutions needed in their specific community helped build the movement. One focus group participant explained, “These are people that I’ve probably sat on committees with, groups with, talking for years and years…This group is actually doing the action behind the talk” (County 4).

Participants reported that greater structure and clarity was needed to support their action steps, particularly among Sector Action Team members that did not participate in the Core Team meetings. They wanted more guidance and support for two reasons in particular—to align their work with the goals and activities undertaken by other Sector Action teams, and to bolster resources and accountability for their own teams. Teams also began to experience capacity gaps as *100% New Mexico’s* volunteer-led initiatives worked to shift from identifying needs through the county-wide survey to action. Relying on volunteer capacity, particularly during the COVID-19 pandemic, resulted in uneven progress between sectors in many counties.

*Role of backbone infrastructure.* Regardless of where their local backbone infrastructure was housed, participants elevated the need to further develop this infrastructure. As described by a participant, “There’s no focal point through which all the information flows” (County 1). Participants identified challenges that included articulating a set of steps to engaging potential partners and community members to sustaining momentum and direction between monthly Sector Team meetings. Participants commented that the current diffusion of administrative duties posed challenges for archiving process-related discussions and decisions for future use.

Participants emphasized that community resources, particularly city and county investments, were critical to establishing needed backbone infrastructure and accountability. As one survey respondent commented, “Currently we are playing off of social capital and personal connection. It is a great start with tons of potential. We have built tons of momentum and can continue, provided the right resources.” Respondents emphasized the need to make purchases such as data systems or portions of staff time. Many initiative members viewed support through their local government agencies as indicators of the buy-in that would be critical to long-term sustainability.

**Inclusivity and partnership.** In contrast to the limited inclusivity and power dynamics that are common critiques of CI, participants drew connections between the community-based coalition approach and their successes. However, despite the high level of agreement that their local CI initiative was broadly inclusive (Fig. 3), many participants discussed the importance of specific strategies to bring particular local government agencies and higher education institutions to the table.

Participants expressed needing strategies for external communication to bolster inclusivity. They reported wanting to continue and expand outreach to the community to bolster participation, particularly from community members. Multiple participants cited raising the visibility of the local CI initiative as a big challenge to their efforts to engage more service providers, the business sector, city and county agencies, and other key community partners. Participants also identified informing state- and administrative-level policy change as a critical communication opportunity. This included strategies to communicate the critical needs of very rural areas to secure crisis housing and other resources where state responses has historically been “your population isn’t big enough for us to justify putting money into your county,” (County 8).

## Discussion

In eight counties implementing *100% New Mexico’s* CI model, we found that the cross-sector framework acted as a strength and a challenge. Participants consistently reported that the cross-sector vision was unique in its approach to transforming the negative SDOH experienced by residents into positive ones. On one hand, the emphasis on de-siloing access to services and reaching across sectors resonated with local CI initiative members and served as an anchor for assessing community member needs and identifying promising strategies. The cross-sector emphasis allowed these local CI initiatives to complement other sector-specific initiatives in their communities, particularly once the local vision became clarified. On the other hand, the sectors varied in their degree of engagement and initiative participation. This was particularly visible at the level of Sector Action Teams, where some teams remained under development as the initiative closed out its first year. This tended to be more common among sectors that had not traditionally been active in public health or other collective impact initiatives. Like O’Neill’s (2020) [23] finding that organizational identity plays a critical role in shaping engagement and the CI approach, our findings suggest that individual sector identities may influence engagement and the CI approach. There may be particular opportunities for this and other CI initiatives to further leverage community organizing approaches [12], particularly to enhance practices in cross-sector collaboration.

The findings demonstrated that supporting local champions to develop their initiatives by leveraging county assets, relationships, and supports, including conducting a county-wide survey and applying results, contributed to progress in mindsets to one of collective action. Counties often made strong progress through conducting the county-wide survey and engaging local leaders and community around the results; across more urban and rural counties, progress slowed at the point of determining what strategies to pursue. In part, the consistency of this experience shines a light on the limits of volunteer capacity. The progress achieved is particularly striking given that this local CI work is primarily conceptualized and led by local volunteers; this departs from the more traditional model of projects initiated via time-limited or sector-specific grants or efforts that emphasis fidelity to a standard approach. However, the findings also suggest that continued progress may require establishing dedicated staff positions or finding other ways to resource the focused project management needed to bring ambitious strategies to fruition, which may be particularly challenging in more rural contexts where capacity and resources have historically been scarce.

Also notable were the participants’ reflections on the sense of partnership and inclusivity. Our findings contrast with a common critique in the growing CI evidence base. In considering what factors may distinguish the *100% New Mexico* experience from other CI initiatives, the framework’s flexibility around the specific organization and structures were appealing according to many. At the same time, there was also widespread recognition that key stakeholders were not yet at the table, both with respect to particular sectors and with regard to the representation and co-leadership of marginalized communities and community members facing the service gaps and barriers that *100% New Mexico* seeks to solve.

## Conclusion

Collective impact is a type of cross-sector partnering to harness the strengths of multiple organizations and community members towards a common agenda. In the case of issues that are complex and multiply determined, applying CI should magnify impact on desired outcomes. Similar to other research, this study found that strong foundational elements of CI were implemented among the eight communities. This included identifying community backbone organizations; engaging a wide range of participants and community leaders, including visual artists and musicians, who committed to a common agenda; and mobilizing teams across the state to collect and review community data to then target solutions to service barriers. Participants resonated with the Anna, Age Eight and 100% Community books and felt that the structure of sector action teams and focus on access to vital services for 100% of New Mexicans fit their communities, particularly during the COVID-19 pandemic. Clear strengths of CI reflected in this study aligned with three of the five strategies for centering equity that are part of CI’s 2022 evolution [11]. There is grounding of the work in data; agreement among participants that the focus is on systems change and collectivity in action; and shaping of the work by communities, as evinced by mural projects and book clubs that have grown into key features of 100% New Mexico. The county-wide survey represents shared measurement, feeding into communities’ use of CQI that includes re-administering the survey over time to assess progress. Through the county-wide survey, we did not find challenges experienced in other applications of CI stemming from not having relevant, available, and timely data, or tension between funder-driven and community-driven desired outcomes [14].

Similar to other research [20], counties in this study varied in the level of satisfaction reported with the core CI component continuous communication. Barata-Cavalcanti, in their assessment of five CI program sites (2020), found that communication strategies at the outset of CI tended to be too simplistic and not focused sufficiently on communication that promotes mutually reinforcing activities. Research is lacking concerning effective communication strategies in CI specifically, but research on communication in other coalitions demonstrates that communication quality impacts coalition effectiveness [24, 25]. This is a key takeaway from the current work; CI implementers may need extra support to anticipate communication needs and implement effective and feasible strategies. In the current study, strong communication may have been lacking initially in several counties because there were few resources to support CI leaders at the time and may also relate to professional development needs or specialized expertise needed. Subsequently, several counties increased utilization of social media such as Facebook and Twitter, began county webpages with the support of the AAEI, and developed shared calendars of county 100% activities. Websites, in particular have been used to share county-wide survey results.

Overall, our results point to a 6 month to 1 year launch process in communities to develop CI foundational infrastructure to address SDOH, within which communication, resources, and engaging the right partners present the most opportunities to strengthen implementation.

## Electronic supplementary material

Below is the link to the electronic supplementary material.

Supplementary Material 1

## Data Availability

Data are not publicly available. They may be available on reasonable request, subject to approval from the ethics committee that approved the study.

## List of Abbreviations

AAEI: Anna, Age Eight Institute
ACES: Adverse Childhood Experiences
CI: Collective Impact
COVID-19: Coronavirus disease of 2019
CQI: Continuous Quality Improvement
SDOH: Social determinants of health
U.S.: United States

## References

[1] Halfon N, Russ SA, Schor EL. The emergence of life course intervention research: Optimizing Health Development and Child Well-Being. Pediatrics. 2022;149(5):e2021053509C. 10.1542/peds.2021-053509C. 10.1542/peds.2021-053509C PMC9847410 35503314

[2] Barton AW, Yu T, Gong Q, Miller GE, Chen E, Brody GH (2022). Childhood poverty, immune cell aging, and African Americans’ insulin resistance: a prospective study. Child Dev.

[3] Janusek LW, Tell D, Gaylord-Harden N, Mathews HL (2017). Relationship of childhood adversity and neighborhood violence to a proinflammatory phenotype in emerging adult african american men: an epigenetic link. Brain Behav Immun.

[4] Larrabee Sonderlund A, Charifson M, Schoenthaler A, Carson T, Williams NJ (2022). Racialized economic segregation and health outcomes: a systematic review of studies that use the index of concentration at the Extremes for race, income, and their interaction. PLoS ONE.

[5] *Social Determinants of Health* Healthy People 2030. U.S. Department of Health and Human Services, Office of Disease Prevention and Health Promotion. https://health.gov/healthypeople/objectives-and-data/social-determinants-health. Accessed 10 Oct 2022.

[6] Courtney KO, Cappello D. Anna, Age Eight: the data-driven prevention of childhood trauma and maltreatment. CreateSpace Independent Publishing Platform; 2018.

[7] Courtney KO, Cappello D. 100% Community: ensuring trauma-free and thriving children, students, and families. CreateSpace Independent Publishing Platform; 2019.

[8] Kania J, Kramer M (2011). Collective impact. Stanf Social Innov Rev.

[9] *StriveTogether 2019 Annual Report: Proving it’s possible place by place*. StriveTogether. 2019 https://www.strivetogether.org/wp-content/uploads/2020/06/2019-StriveTogether-Annual-Report_D6.pdf. Accessed 10 Oct 2022.

[10] Ennis G, Tofa M (2020). Collective impact: a review of the peer-reviewed research. Australian Social Work.

[11] Kania J, Williams J, Schmitz P, Brady S, Kramer M, Juster JS (2022). Centering equity in collective impact. Stanf Social Innov Rev.

[12] Christens BD, Inzeo PT (2015). Widening the view: situating collective impact among frameworks for community-led change. Community Dev.

[13] Abresch C, Grimm B, Lyons K, Maloney S, Tibbits M (2022). Who gets included in collective impact: a mixed methods study of 10 CI initiatives. Community Dev.

[14] Dolamore S, Kline A (2020). Strengthening community-based health and human services in the shadow of structural inequality: a critical case study of the collective impact model. J Health Hum Serv Adm.

[15] Bradley K, Chibber KS, Cozier N, Meulen PV, Ayres-Griffin C (2017). Building Healthy Start Grantees’ capacity to achieve collective impact: Lessons from the field. Matern Child Health J.

[16] Gwynne K, Cairnduff A (2017). Applying collective impact to wicked problems in Aboriginal health. Metropolitan Universities Journal.

[17] Patton MQ. Developmental evaluation: applying complexity concepts to enhance innovation and use. Guilford Press; 2010.

[18] Preskill H, Parkhurst M, Juster JS. Learning and evaluation in the collective impact context. *Guide to evaluating collective impact*. 2001. https://collectiveimpactforum.org/sites/default/files/Guide%20to%20Evaluating%20CI. Accessed 10 Oct 2022.

[19] Ingram RC, Scutchfield FD, Mays GP, Bhandari MW (2012). The economic, institutional, and political determinants of public health delivery system structures. Public health reports (Washington D C : 1974).

[20] Barata-Cavalcanti O, Leung MM, Costa S, Mateo KF, Guillermin M, Palmedo CP, Crossley R, Huang TT (2020). Assessing the collective impact of Community Health Programs funded by Food and Beverage Companies: a New Community-Focused methodology. Int Q Community Health Educ.

[21] Braun V, Clarke V. Conceptual and design thinking for thematic analysis. Qualitative Psychol. 2022 Feb;9(1):3.

[22] Miles MB, Huberman AM, Saldaña J. Qualitative data analysis: a methods sourcebook. Sage publications; 2018.

[23] O’Neill M (2020). Increasing community engagement in collective impact approaches to advance social change. Community Dev.

[24] Brown LD, Wells R, Jones EC, Chilenski SM (2017). Effects of Sectoral Diversity on Community Coalition processes and outcomes. Prev science: official J Soc Prev Res.

[25] Wagner CL, Fernandez-Gimenez ME (2009). Effects of community-based collaborative group characteristics on social capital. Environ Manage.

