# Supplementary material for: Advancing positive social determinants of health through collective impact and the 100% New Mexico Model
Source: Arch Public Health. 2023 Jun 16;81:109. doi: 10.1186/s13690-023-01120-4 (PMC10273763; doi:10.1186/s13690-023-01120-4)
Supplement: Supplementary file 1 — Supplementary Material 1 [file 13690_2023_1120_MOESM1_ESM.docx]

**Aims and Scope Statement**

**Advancing positive social determinants of health through collective impact**

Collective impact (CI) as an approach to solving complex problems is a relatively new approach, developed in 2011 and adopted internationally in various contexts to address a range of health issues. The predominance of model application and its research to date tends to be issue-specific. That is, implementers use CI to address a specific health issue such as smoking or obesity. The CI research we are conducting is to determine CI’s relevance and ability to influence outcomes in social determinants of health (SDOH), which in its root definition is preventive and involves the contribution of multiple service sectors and government actors. The study of CI to address SDOH is a growing research area that is currently mostly developmental but will likely increase in rigor as emerging models, such as the one studied here, are sustained and develop shared data systems (e.g. John et al., 2021).

Our study adds to what is known about the cultural relevance of CI, and the core strategies that were developed locally and community-driven to mobilize CI in this context. Our study found new key partners integrated into CI that may have to do with having SDOH as the umbrella goal compared with previous issue-centric efforts. We also did not find challenges experienced in other applications of CI that stemmed from a lack of relevant, available, and timely data, or tension between funder-driven and community-driven desired outcomes. We believe this has to do with this particular initiative’s approach in “starting with data” through community-administered surveys that not only helped implementers identify solutions to identified problems but created ownership and a sense of collective efficacy that may portend sustainability if issues of available resources are also addressed.

Our study found that more sophisticated communication channels and supports were needed during the launch of this CI model. Local teams were not equipped with the knowledge and skills necessary to anticipate, assess, and resolve issues with a lack of communication across their work teams. This is a key study takeaway and one that future CI SDH initiatives should consider being proactive about early in implementation.

**Reference** [John et al. (2021). Building and advancing coalition capacity to promote health equity: Insights from the Health Equity Collective's Approach to Addressing Social Determinants of Health](https://www.ncbi.nlm.nih.gov/pmc/articles/PMC8742293/)
